# Acute effects of concentric and eccentric exercise matched for energy expenditure on glucose metabolism in healthy females: a randomized crossover trial

**DOI:** 10.1186/s40064-016-3062-z

**Published:** 2016-08-30

**Authors:** Marc Philippe, Georg Junker, Hannes Gatterer, Andreas Melmer, Martin Burtscher

**Affiliations:** 1 Department of Sport Science, Medical Section, University of Innsbruck, Fürstenweg 185, 6020 Innsbruck, Austria; 2 Department of Internal Medicine I, Medical University of Innsbruck, Anichstraße 35, 6020 Innsbruck, Austria

**Keywords:** Concentric exercise, Eccentric exercise, Glucose metabolism, Interleukin 6, Tumor necrosis factor alpha

## Abstract

**Background:**

Single bouts of muscle damaging eccentric exercise (EE) affect glucose metabolism negatively while single bouts of concentric (CE) and not muscle damaging eccentric exercise have positive acute short-term effects on glucose metabolism. It has been proposed that long-term endurance EE might be more effective in improving glucose metabolism than long-term CE when adjusted for energy expenditure. This would imply that adaptations of glucose metabolism are dependent on the type of exercise. Interleukin-6 (IL-6) released from the exercising muscles may be involved in and could therefore explain acute adaptations on glucose metabolism. The aim of the study was to investigate the effects of a single bout of CE and a single bout of EE inducing no or just mild muscle damage, matched for energy expenditure, on glucose metabolism.

**Methods:**

7 healthy but sedentary female participants (age 20.7 ± 2.9 years; BMI 22.45 ± 1.66 kg m^−2^; VO_2_peak 39.0 ± 4.5 ml kg^−1^ min^−1^) took part in a randomized cross over trial consisting of 1 h uphill (CE) respectively downhill (EE) walking on a treadmill. Venous blood samples were drawn before, directly after and 24 h after exercise. An oral glucose tolerance test (OGTT) was performed before and 24 h after exercise.

**Results:**

CE and EE lead to comparable changes of glucose tolerance (area under the curve of the OGTT) (−16.0 ± 25.81 vs. −6.3 ± 45.26 mg dl^−1^ h^−1^, *p* = 1.000) and HOMA insulin resistance (−0.16 ± 1.53 vs. −0.08 ± 0.75, *p* = 0.753). Compared to baseline, IL-6 concentration increased significantly immediately after EE (1.07 ± 0.67 vs. 1.32 ± 0.60 pg ml^−1^, *p* = 0.028) and tended to increase immediately after CE (0.75 ± 0.29 vs. 1.03 ± 0.21 pg ml^−1^, *p* = 0.058). TNF-α concentration decreased significantly immediately after EE (1.47 ± 0.19 vs. 1.06 ± 0.29 pg ml^−1^, *p* = 0.046) but not after CE (1.27 ± 0.43 vs. 1.24 ± 0.43 pg ml^−1^, *p* = 0.686) compared to baseline.

**Conclusions:**

Acute effects of a single bout of exercise inducing no or just mild muscle damage on glucose tolerance and insulin resistance seem to be primarily energy expenditure dependent whereas acute anti-inflammatory activity induced by a single bout of exercise appears to be rather exercise type dependent.

*Trial registration*: NCT01890876, clinicaltrials.gov, https://clinicaltrials.gov/.

## Background

Performing concentric (muscle shortening contractions; CE) and eccentric (muscle lengthening contractions; EE) endurance exercises on a regular basis has been suggested to be similarly effective in improving glucose metabolism in sedentary healthy subjects (Drexel et al. 2008; Zeppetzauer et al. 2013). When considering calculated energy expenditure, the adaptations of glucose tolerance to EE seem to be even superior as compared to those elicited by CE. This might indicate an exercise type dependency (Zeppetzauer et al. 2013). However, these findings remain controversial (Marcus et al. 2009) and physiological explanations are missing.

Single bouts of concentric endurance exercise elicit similar acute short-term effects on glucose metabolism as long-term CE and are generally associated with improved insulin action and glucose transport (Wojtaszewski et al. 2002). However, muscle damage induced by an unaccustomed bout of eccentric exercise may negatively affect glucose metabolism (Kirwan and del Aguila 2003). Nevertheless, Philippe et al. (2016) showed that a single bout of non-muscle-damaging EE can positively influence glucose tolerance. Yet, no mode dependency was found as the effects on glucose metabolism were predominantly influenced by energy expenditure but not by exercise type (Philippe et al. 2016). These results indicate that by avoiding muscle damage, single bouts of CE and EE would elicit similar acute short-term effects on glucose metabolism when matched for energy expenditure. The acute short-term EE effects on glucose metabolism may also reflect long-term adaptations as was already shown for CE.

Interleukin 6 (IL-6) released by muscle contraction (Steensberg et al. 2000) supports the maintenance of metabolic homeostasis during exercise (Febbraio and Pedersen 2002). Moreover, IL-6 concentrations after non-muscle-damaging exercise could be a direct indicator for GLUT4 translocation and may therefore help to explain changes in glucose metabolism after exercise (Carey et al. 2006). In addition, muscle derived IL-6 inhibits the production of the pro-inflammatory cytokine tumor necrosis factor alpha (TNF-α) (Petersen and Pedersen 2005; Starkie et al. 2003). This acute exercise induced anti-inflammatory effect may be exercise type dependent, favoring EE over CE (Philippe et al. 2016).

To the best of the authors’ knowledge, no study investigated the effects of a single bout of CE and a single bout of EE inducing no or just mild muscle damage, matched for energy expenditure, on glucose metabolism and anti-inflammatory responses. However, this would be interesting to further investigate as acute short-term effects on glucose metabolism may also reflect long-term adaptations and may therefore clarify if EE could actually be considered more effective as deducted from calculations (Zeppetzauer et al. 2013).

Based on the above, we hypothesize that a single bout of CE and a single bout of EE inducing no or just mild muscle damage have similar positive acute short-term effects on glucose metabolism but different anti-inflammatory effects.

**Fig. 1:**
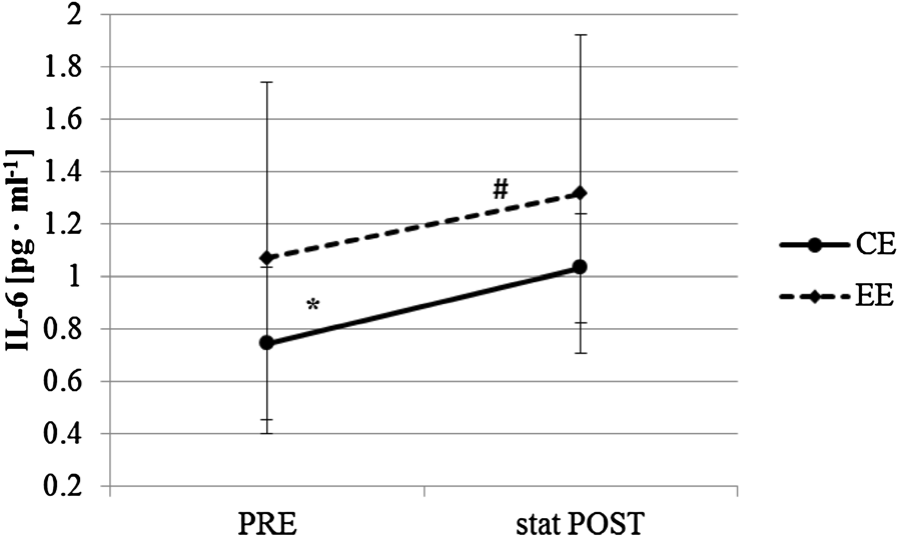
Immediate changes of interleukin 6 (IL-6) concentration (#significant change within EE; *tendential change within CE); baseline = PRE, immediately after exercise = stat POST

**Fig. 2:**
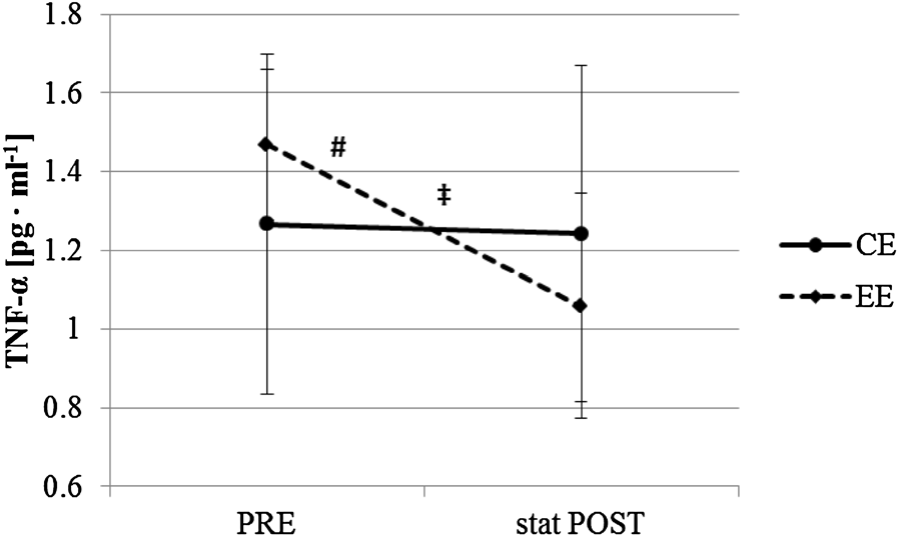
Immediate changes of tumor necrosis factor alpha (TNF-α) concentration (^#^ significant change within EE; ^**‡**^significant interaction between CE and EE); baseline = PRE, immediately after exercise = stat POST

## Methods

### Study participants

Based on the results reported by Philippe et al. (2016), a total sample size of N = 6 for >80 % power has been calculated for changes in glucose tolerance due to CE (G*Power, Version 3.1.5). Seven young and healthy females volunteered to participate in the study. Each participant underwent medical routine examination including medical history. The subjects had to match following criteria: female sex, being sedentary (less than 2.0 h of physical exercise per week), non-smoker, no acute or chronic diseases that would hamper the safe performance of exercise tests, and must not be accustomed to eccentric training. Characteristics of the study participants are shown in Table 1. Written informed consent was provided by every participant. The study was approved by the institutional review board of the Department of Sport Science of the University of Innsbruck. All study procedures comply with the declaration of Helsinki 1964 and its later amendments.

**Table 1:** Characteristics of the study participants (N = 7)

	Mean ± SD
Age (years)	20.7 ± 2.9
Height (m)	1.70 ± 0.06
Body mass (kg)	60.0 ± 11.8
BMI (kg m^−2^)	22.5 ± 1.7
Hfpeak (beats min^−1^)	192.3 ± 7.8
VO_2_peak (ml kg^−1^ min^−1^)	39.0 ± 4.5

### Study protocol

The study was designed as a randomized crossover trial. On a pre-test day subjects underwent routine examination followed by a maximal incremental treadmill exercise. One week later (PRE) between 7:30 a.m. and 9:30 a.m., the subjects appeared in a fasting state (minimum 10 h without food intake and water as the only fluid) and underwent venous blood sampling from the antecubital vein and an oral glucose tolerance test (OGTT). This was followed by 1 h of either eccentric (going downhill—EE) or 1 h of concentric (going uphill—CE) treadmill exercise. The participants were not allowed to eat between the OGTT and the 1 h exercise or during the 1 h exercise. However, they were allowed to drink water during the 1 h exercise. Immediately after finishing the walking exercise (stat POST), venous blood samples were taken again. 24 h later (24 h POST), the subjects appeared again in a fasting state and underwent venous blood sampling and an oral glucose tolerance test (OGTT). Two weeks later, the subjects completed the same protocol with the opposite exercise type. The participants were asked to pursue their normal eating habits during the study phase.

To avoid muscle damage that could hamper potential beneficial effects of EE (Kirwan et al. 1992; Pokora et al. 2014), a treadmill slope of +14 % for CE and −14 % for EE and a walking speed that was adapted to match an exercise intensity corresponding to 40 % of the VO_2_max was chosen. The energy expenditure was controlled by gas analysis after 15, 30 and 45 min during the walking trials and walking speed was adapted if necessary. The average walking speed for CE was 0.80 ± 0.10 m s^−1^ and for EE 1.67 ± 0.13 m s^−1^.

The exercise capacity tests and the walking tests were performed on the same treadmill in the same room during January and February 2015. The mean ambient room temperature was 22 ± 1 °C.

### Exercise capacity

Exercise capacity was assessed on a treadmill (Pulsar, h/p/cosmos, Nussdorf-Traunstein, Germany) by using respiratory gas analysis (Oxycon Mobile, Viasys Healthcare, Hoechberg, Germany). The test began at a velocity of 2 km h^−1^ and an elevation of 10 % for 2 min followed by 2 min at the same speed at 12 % of elevation and 1 min at the same speed at 14 % of elevation. After that, the elevation remained unchanged and the velocity increased by 0.5 km h^−1^ each minute until exhaustion or limitation by symptoms.

### Measurements of glucose metabolism, IL-6, TNF-α

Venous blood samples were used to assess insulin sensitivity PRE and 24 h POST by using the homeostasis model assessment [HOMA = fasting insulin (μU ml^−1^) fasting glucose (mg dl^−1^)/405]. Additionally IL-6 and TNF-α were determined PRE, stat POST and 24 h POST. All parameters were assessed using standard, state of the art techniques at the laboratory of the Medical University of Innsbruck. Plasma glucose was quantified using a commercially available enzymatic kit (Roche Diagnostic Systems, Basel, Switzerland) on a Hitachi 902 autoanalyzer (Roche Diagnostic Systems, Basel, Switzerland). Insulin was determined using automated analyzers within the central clinical laboratory at the Medical University of Innsbruck. IL-6 and TNF-α were both determined using ELISA kits commercially available (R&D systems, Minneapolis, Minnesota, USA) following the manufacturer’s instructions. Glucose tolerance was assessed PRE and 24 h POST via an oral glucose tolerance test (OGTT). Participants had to drink 75 g glucose dissolved in 300 ml water. Capillary blood samples were drawn and analyzed before (OGTT0) and 1 h (OGTT1) and 2 h (OGTT2) after ingestion (Biosen C-Line, EKF-Diagnostics, Germany). The area under the curve (AUC) of the OGTT was calculated with the trapezoidal rule as proposed by Le Floch et al. (1990).

### Measurement of muscle damage and delayed onset muscle soreness (DOMS)

Creatine kinase (CK) as an indirect indicator for muscle damage was assessed from capillary blood at the time points PRE and 24 h POST (Reflotron Sprint, Boehringer Mannheim, Mannheim, Germany). 24 h POST CE and EE DOMS was assessed via a graded scale for muscle-pain and muscle-soreness respectively, ranging from 1 to 10 (1 meaning no pain/soreness and 10 meaning maximal pain/soreness).

### Statistical analysis

Due to the small sample size non-parametric statistical tests were used (IBM, SPSS Statistics, Version 20). Changes from PRE to stat POST respectively 24 h POST within each group were calculated with Wilcoxon Matched Pairs Test. Interactions (time × group) between CE and EE were computed from the differences of stat POST respectively 24 h POST and PRE values with Wilcoxon Matched Pairs Test. Relations between changes of glucose metabolism and acute concentrations changes of IL-6 were calculated with Spearman’s rank correlation coefficient. Differences were considered statistically significant at *p* ≤ 0.05.

## Results

All baseline values (PRE) values did not differ significantly between CE and EE.

### Glucose metabolism and inflammatory parameters PRE CE/EE to 24 h POST CE/EE

Changes of all baseline metabolic and inflammatory parameters compared to 24 h post-exercise are presented in Table 2. OGTT0 tended to improve 24 h POST CE but not 24 h POST EE. Overall glucose tolerance (AUC) and insulin resistance (HOMA) had slightly improved 24 h POST CE and EE but did not reach statistical significance for both trials. No other metabolic or inflammatory parameters were significantly modified 24 h POST CE or EE, respectively. There was a significant interaction for OGTT0 between CE and EE.

**Table 2:** Baseline (PRE) and 24 h after exercise (24 h POST) metabolic and inflammatory parameters after 1 h of uphill (CE) and downhill (EE) walking at the same energy expenditure

	PRE CE mean ± SD	24 h POST CE mean ± SD	Within *p* values	PRE EE mean ± SD	24 h POST EE mean ± SD	Within *p* values	Interaction *p* values
OGTT0 (mg dl^−1^)	82.8 ± 11.8	74.2 ± 12.9	0.091	79.1 ± 13.7	85.0 ± 6.3	0.128	0.018
OGTT1 (mg dl^−1^)	114.0 ± 28.8	104.2 ± 19.4	0.128	118.2 ± 23.2	109.4 ± 15.1	0.499	0.735
OGTT2 (mg dl^−1^)	91.5 ± 16.4	87.8 ± 14.3	1.000	100.3 ± 18.9	99.5 ± 11.1	1.000	0.735
AUC (mg dl^−1^ h^−1^)	201.2 ± 31.7	185.2 ± 26.9	0.128	207.9 ± 35.5	201.6 ± 17.0	0.499	1.000
Insulin (mU l^−1^)	9.45 ± 3.87	8.80 ± 5.23	0.674	7.23 ± 3.06	6.63 ± 2.72	0.917	0.917
HOMA	2.18 ± 0.97	2.02 ± 1.17	0.753	1.63 ± 0.63	1.55 ± 0.63	0.917	0.753
IL-6 (pg ml^−1^)	0.75 ± 0.29	0.88 ± 0.23	0.173	1.07 ± 0.67	0.86 ± 0.26	0.917	0.249
TNF-α (pg ml^−1^)	1.27 ± 0.43	2.17 ± 1.78	0.600	1.47 ± 0.19	3.36 ± 3.82	0.173	0.917
CK (U l^−1^)	117.8 ± 63.6	97.0 ± 42.0	0.172	98.8 ± 61.5	197.2 ± 58.5	0.046	0.028

*CE* concentric exercise, *EE* eccentric exercise, *OGTT* oral glucose tolerance test, *OGTT0* fasting glucose concentration in capillary blood, *OGTT1* glucose concentration in capillary blood 1 h after drinking 75 g of glucose dissolved in 300 ml of water, *OGTT2* glucose concentration in capillary blood 1 h after drinking 75 g of glucose dissolved in 300 ml of water, *AUC* area under the curve of the oral glucose tolerance test, *HOMA* homeostasis model assessment of insulin resistance, *IL*-*6* interleukin 6, *TNF*-*α* tumor necrosis factor alpha, *CK* creatine kinase; within group changes were calculated with Wilcoxon test, interactions (time × group) were calculated from the mean differences with Wilcoxon test

### Assessment of muscle damage and delayed onset muscle soreness (DOMS)

EE resulted in mild muscle damage. CK concentrations were significantly higher 24 h POST than PRE EE (Table 2) and pain and soreness scores were significantly higher 24 h POST EE than 24 h POST CE (3.1 ± 2.4 vs. 1.0 ± 0.0, *p* = 0.042; 2.6 ± 2.4 vs. 1.0 ± 0.0, *p* = 0.034).

### IL6 and TNF-α concentration changes immediately after CE and EE

IL-6 concentration increased significantly from PRE to stat POST EE (1.07 ± 0.67 vs. 1.32 ± 0.60 pg ml^−1^, *p* = 0.028) and tended to increase stat POST CE (0.75 ± 0.29 vs. 1.03 ± 0.21 pg ml^−1^, *p* = 0.058). There was no interaction between CE and EE (*p* = 0.753) (Fig. 1). TNF-α concentration decreased significantly from PRE to stat POST after EE (1.47 ± 0.19 vs. 1.06 ± 0.29 pg ml^−1^, *p* = 0.046) but not stat POST CE (1.27 ± 0.43 vs. 1.24 ± 0.43 pg ml^−1^, *p* = 0.686). There was a significant interaction between CE and EE (*p* = 0.046) (Fig. 2).

#### Correlation analysis

We found a significant negative correlation between insulin concentration changes (24 h POST minus PRE) and immediate IL-6 concentration changes (stat POST minus PRE) for CE (R = −0.886, *p* = 0.019).

## Discussion

The main findings of this study are as follows. First, single bouts of CE or EE with the same energy expenditure led to comparable acute short-term effects on glucose tolerance and insulin resistance, indicating an energy expenditure dependency and not exercise type dependency. Secondly, TNF-α production was inhibited stronger immediately after EE compared to CE assuming an exercise type dependency of this acute exercise induced anti-inflammatory reaction.

Although the acute effects on glucose tolerance and insulin resistance were not statistically significant, CE as well as EE led to metabolic changes in a positive direction. This is in contrast to the common findings in literature reporting acute negative effects of EE on glucose metabolism, especially transient insulin resistance, non-insulin dependent glucose uptake and muscle glycogen replenishment (Asp et al. 1996; del Aguila et al. 2000; Doyle et al. 1993; Green et al. 2010; Ide et al. 1996; Kirwan et al. 1992; Sherman et al. 1992). Our findings confirm that even a single bout of EE can provoke positive acute short-term effects on glucose metabolism (Philippe et al. 2016). We did not completely achieve our goal of avoiding muscle damage. Despite choosing a low slope gradient and a low workload, some participants may have experienced muscle damage after EE that may have masked additional positive effects. However, after EE the change of AUC and the change of CK did not correlate (R = 0.368; *p* = 0.473). Therefore, we may assume that muscle damage was mild and not interfering with glucose metabolism at the time point of our POST measurement.

Our results are different to the results of Zeppetzauer et al. (2013) who reported a higher effectiveness of long-term EE in improving glucose tolerance compared to long-term CE when adjusted for energy expenditure. When considering that single bouts of concentric endurance exercise elicit similar acute short-term effects on glucose metabolism as long-term CE (Wojtaszewski et al. 2002) and that EE may elicit the same acute short-term positive effects when muscle damage is avoided (Philippe et al. 2016), our findings give a strong indication that adaptations of glucose metabolism seem to be energy expenditure dependent and not exercise type dependent.

The immediate changes of IL-6 concentrations after CE and EE even strengthen our assumption of an energy dependency as IL-6 is considered as an energy sensor of the muscle (MacDonald et al. 2003; Pedersen 2012). While we could show in our previous study that the immediate rise of IL-6 was significantly higher stat POST CE compared to EE, where CE and EE were not adjusted for energy expenditure (Philippe et al. 2016), IL-6 concentration increased nearly identically directly after CE and EE when adjusted for energy expenditure. Thus, the overall metabolic cost influenced by exercise intensity, time and muscle mass involved is assumed to be the major trigger for IL-6 release and the related metabolic adaptations to exercise (Pedersen and Febbraio 2008).

The negative correlation between immediate concentration changes of IL-6 (stat POST minus PRE) and short-term insulin concentration changes (24 h POST minus PRE) are an indication for an improved insulin sensitivity after CE as it was shown that IL-6 produced by the exercising muscle enhances insulin stimulated glucose disposal (Carey et al. 2006). The slightly improved insulin sensitivity may explain the slightly improved glucose tolerance 24 h POST CE. However no such correlation could be found after EE. It may be assumed, that despite having exercised at the same energy expenditure, EE induced higher strain on the working muscles (Camillo et al. 2015; Johnson et al. 2002), as indicated by muscle damage and may therefore be more comparable to a resistance endurance training than to aerobic exercise. This would favor insulin independent GLUT4 translocation and glucose transport mediated via IL-6 (Carey et al. 2006) and via a non-energy dependent pathway over the GTPase Rac1 induced by muscle stretching, i.e. mechanical stress (Sylow et al. 2013, 2014, 2015). These mechanisms could explain the slightly improved glucose tolerance found 24 h POST EE.

It was shown that IL-6 secreted by the working muscle during exercise stimulates the appearance of other anti-inflammatory cytokines in the circulation and inhibits the production of the pro-inflammatory cytokine TNF-α (Petersen and Pedersen 2005; Starkie et al. 2003). Previously we showed that CE and EE may lead to acute TNF-α concentration reductions directly after exercise (Philippe et al. 2016). In the present study, where CE and EE were performed at the same energy expenditure, we found concentration reductions immediately after CE and EE but the decrease of TNF-α concentration was only significant immediately after EE and significantly higher immediately after EE compared to CE. This is further evidence that exercise induces acute anti-inflammatory activity but our findings suggest that there is an exercise type dependency of this exercise induced anti-inflammatory reaction.

### Limitations

First of all, the small sample size may be considered to be a weakness of our study which we tried to overcome by the use of a crossover design. Secondly, we could not completely avoid muscle damage after EE. Future studies could avoid muscle damage by exposing their participants to a bout of unaccustomed EE causing muscle damage with reduced muscle function and impaired glucose tolerance prior to testing, as it appears to be protective against further negative effects for 1–4 weeks (Green et al. 2010; Maeo et al. 2015). Thirdly, the overall metabolic cost of our single bouts of CE and EE may have been too low to elicit significant changes to glucose metabolism. In prospect of avoiding muscle damage, this issue could best overcome by choosing longer exercise durations but no higher exercise intensity.

Fourthly, we only measured concentration changes of two cytokines which may not be sufficient to reflect a general inflammatory status. However, as IL-6 is the cytokine produced in the highest quantity directly by the exercising muscle, upregulating other anti-inflammatory cytokines and downregulating TNF-α being a key regulator of inflammatory response (Petersen and Pedersen 2005), IL-6 and TNF-α may be sufficient to describe acute exercise induced anti-inflammatory action. Fifthly, there was a substantial difference of standard deviations between IL-6 PRE CE and IL-6 PRE EE. This originated from one participant who had a relatively high IL-6 concentration PRE EE (2.09 pg ml^−1^ PRE EE vs. 0.92 pg ml^−1^ PRE CE). As TNF-α levels of the same participant were slightly higher PRE EE compared to PRE CE too (1.37 vs. 0.93 pg ml^−1^), there may have been a low-grade inflammation. However, the higher IL-6 level PRE EE did not affect the results as the IL-6 stat POST EE value of the same participant was 2.10 pg ml^−1^.

## Conclusion

Single bouts of CE or EE with the same energy expenditure led to comparable acute short-term changes in glucose tolerance and insulin resistance, indicating an energy expenditure dependency and not exercise type dependency. This assumption is supported by the finding that IL-6 secretion of the working muscle is similar immediately after single bouts of CE and EE when both exercise types are performed at the same energy expenditure. We could confirm that exercise induces acute anti-inflammatory activity but TNF-α concentration decreased more pronounced directly after EE compared to CE assuming an exercise type dependency of this exercise induced anti-inflammatory response.
